# Unscheduled HDAC4 repressive activity in human fibroblasts triggers TP53‐dependent senescence and favors cell transformation

**DOI:** 10.1002/1878-0261.12392

**Published:** 2018-11-14

**Authors:** Harikrishnareddy Paluvai, Eros Di Giorgio, Claudio Brancolini

**Affiliations:** ^1^ Department of Medicine Università degli Studi di Udine Italy

**Keywords:** AKT, class IIa HDACs, MEF2, OIS, pRB, RAS

## Abstract

Expression of the class IIa HDACs is frequently altered in different human cancers. In mouse models these transcriptional repressors can trigger transformation, acting as bona fide oncogenes. Whether class IIa HDACs also exhibit transforming activities in human cells is currently unknown. We infected primary human fibroblasts with retroviruses to investigate the transforming activity of HDAC4 in cooperation with well‐known oncogenes. We have discovered that HDAC4 triple mutant (S246A, S467A, S632A) (HDAC4‐TM), a nuclear resident version of the deacetylase, triggers TP53 stabilization and OIS (oncogene‐induced senescence). Unlike RAS, HDAC4‐induced OIS was TP53‐dependent and characterized by rapid cell cycle arrest and accumulation of an unusual pattern of γH2AX‐positive foci. The inactivation of both TP53 and of the retinoblastoma (pRb) tumor suppressors, as induced by the viral oncogenes large and small T of SV40, triggers anchorage‐independent growth in RAS, HDAC4‐TM and, to a lesser extent, in HDAC4‐wild type (WT)‐expressing cells. Our results suggest an oncogenic function of class IIa HDACs in human cells, and justify further efforts to discover and evaluate isoform‐specific inhibitors of these epigenetic regulators from a therapeutic perspective.

**Abbreviations**
4‐OHT: 4 hydroxy‐tamoxifen
BrdU: bromodeoxyuridine
DDR: DNA damage response
DEG: differentially expressed genes
DMEM: Dulbecco's modified Eagle's medium
DN: dominant negative
EMEM: Earle's salts minimal essential medium
ENG: engrailed
ER: estrogen receptor alpha
GSEA: gene set enrichment analysis
HDAC4‐TM: HDAC4 triple mutant (S246A, S467A, S632A)
HDAC: histone deacetylase
HPRT: hypoxanthine phosphoribosyltransferase 1
IFN: interferon
LT: large T antigen
MEF2: myocyte enhancer factor
MTT: 3‐(4,5‐dimethylthiazol‐2‐yl)‐2,5‐diphenyltetrazolium bromide
myr‐AKT1: myristoylated AKT1
OIS: oncogene‐induced senescence
pRb: retinoblastoma protein
PTM: post‐transcriptional modification
SASP: senescence‐associated secretory phenotype
ST: small T antigen
TERT: telomerase catalytic subunit
TF: transcription factor
WT: wild type

## 1 Introduction

The remodeling of transcriptional programs is a key aspect of tumorigenic processes (Bradner *et al*., 2017). Oncogenes and tumor suppressor genes can influence transcription directly by acting as transcription factors (TFs) or indirectly by supervising signaling pathways that control the transcriptional machinery (Bradner *et al*., 2017). Transcriptional remodeling requires epigenetic changes and the involvement of chromatin modifiers (Brien *et al*., 2016; Yang *et al*., 2012). Epigenetic plasticity is emerging as an important hallmark of cancer. At each step of tumor progression, from initiation to evolution, epigenetic plasticity is necessary to fuel new waves of transcription (Koschmann *et al*., 2017).

Histone modifications contribute to chromatin remodeling and to the epigenetic signature. Dysfunctions in enzymes controlling these post‐transcriptional modifications (PTMs) can affect the tumorigenic process (Cheng *et al*., 2017a,b; Montero‐Conde *et al*., 2017). Both pro‐tumorigenic and tumor‐suppressive activities have been reported for some epigenetic regulators, which reflects the importance of the specific cellular context (Beira *et al*., 2018; Chen *et al*., 2018; Koschmann *et al*., 2017; Wang *et al*., 2017).

In addition to alterations in epigenetic regulators, mutations in histones can trigger the tumorigenic process. Oncogenic mutations in H3.3 genes encoding K27M and G34R/V have been characterized in pediatric high‐grade gliomas (Fontebasso *et al*., 2014; Khuong‐Quang *et al*., 2012; Schwartzentruber *et al*., 2012; Taylor *et al*., 2014).

Histone deacetylases (HDACs) are important erasers of epigenetic marks. HDAC can be subdivided into five subclasses (I, IIa, IIb, III and IV). Class IIa includes four members (HDAC4/5/7/9) characterized by a poor deacetylase activity, phosphorylation‐dependent nuclear/cytoplasmic shuttling and an extended amino‐terminal region devoted to TFs and co‐repressor binding (Martin *et al*., 2007). The C‐terminal deacetylase domain is required to interact with the SMRT/N‐CoR/HDAC3 complex, which confers the KDAC activity (Lahm *et al*., 2007). Important partners of class IIa HDACs are members of the MEF2 (myocyte enhancer factors) family of TFs (Clocchiatti *et al*., 2013a,b; Di Giorgio and Brancolini, 2016). There is evidence from several reports of the altered expression of class IIa in human cancer (Barneda‐Zahonero and Parra, 2012; Clocchiatti *et al*., 2011). Furthermore, *in vitro* and *in vivo* studies have proved the oncogenic role of HDAC4 (Di Giorgio *et al*., 2013; Peruzzo *et al*., 2016), HDAC7 (Di Giorgio *et al*., 2013; Lei *et al*., 2017; Rad *et al*., 2010) and HDAC9 (Gil *et al*., 2016) in different murine models. Since human cells behave differently from rodent cells and, in general, require more genetic alterations to acquire a neoplastic phenotype (Boehm *et al*., 2005), whether these deacetylases can transform human cells is currently unknown.

*In vitro* oncogenic transformation of normal cells represents an invaluable model to prove tumor‐suppressive or oncogenic functions of a specific gene (Funes *et al*., 2014). The robustness of the assay is corroborated by the correlation between the *in vitro* transforming activities of the tested genes and their implications in human cancers (Boehm and Hahn, 2005; Boehm *et al*., 2005; Maya‐Mendoza *et al*., 2015; Seger *et al*., 2002). Moreover, these *in vitro* generated transformed cells can provide alternatives to costly mouse models, as well as genetically defined environments for testing anticancer therapies (Balani *et al*., 2017). In this manuscript we investigated the ability of HDAC4 to cooperate with well‐known oncogenes to transform human fibroblasts.

## 2 Materials and methods

### 2.1 Cell cultures and reagents

The BJ/TERT cells were cultured in Earle's salts minimal essential medium (EMEM) (Euroclone, Milan, Italy) completed with nonessential amino acids (HyClone, Little Chalfont, UK). Media were supplemented with 10% FBS, L‐glutamine (2 mm), penicillin (100 U·mL^−1^), and streptomycin (100 μg·mL^−1^) (Lonza, Basel, Switzerland). Cells expressing the inducible form of the transgenes were grown in medium without phenol red. For S‐phase analysis, cells were grown for 3 h with 50 μm bromodeoxyuridine (BrdU, Sigma). Mouse anti‐BrdU (Sigma, St. Louis, MO, USA) was used as the primary antibody in the immunofluorescent assays. Nuclei were stained with Hoechst 33258 (Sigma).

### 2.2 Invasion and soft agar assays

For invasion assay, each well of the invasion chamber (CLS3428, Corning, New York, NY, USA) was coated with 200 μL of Matrigel matrix coating solution (Cultrex, Trevigen, Gaithersburg, MD USA). Next, a cell suspension of 30 000 cells in 0.1% FBS‐Dulbecco's modified Eagle's medium (DMEM) was added. As chemoattractant, 20% FBS‐DMEM was added in each lower chamber. As a control, 0.1% FBS‐DMEM was used to evaluate random invasion. For soft agar assays, equal volumes of 1.2% agar and DMEM were mixed to generate 0.6% base agar. A total of 0.8 × 105 cells were seeded in 0.3% top agar, followed by incubation at 37 °C in humidified conditions. The cells were grown for 21 days and visualized by MTT [3‐(4,5‐dimethylthiazol‐2‐yl)‐2,5‐diphenyltetrazolium bromide] staining. Images were taken with a DN6000 Leica microscope that allows the the number and the diameter of the foci to be counted.

### 2.3 Plasmid construction, transfections, retroviral infections

The pWZL/HDAC4‐WT and mutants, pWZL‐Neo/E1A (1–143) and pBABE‐Puro/myristoylated AKT1 (myr‐AKT1)‐expressing plasmids were previously described (Astle *et al*., 2012; Deng *et al*., 2005; Di Giorgio *et al*., 2013). pCW‐Puro/HDAC4 and H‐RAS/G12V plasmids were obtained by subcloning with a PCR method, HDAC4 and H‐RAS/G12V into the all‐in‐one DOX‐inducible pCW‐Cas9 plasmid (#50661 Addgene, Cambridge MA, USA). pMXPIE‐Puro HDAC4‐WT/TM, H‐RAS/G12V were obtained by subcloning with a PCR method the ORF into the 4 hydroxy‐tamoxifen (4‐OHT)‐ inducible pMXPIE plasmid (Toledo *et al*., 2008). The plasmids used to silence MEF2D and plasmids encoding for MEF2D‐FLAG and MEF2‐Engrailed FLAG (MEF2‐ENG) were previously described (Di Giorgio *et al*., 2015, 2017). For lentivirus‐based knock‐down, HEK‐293T cells were transfected with 1.8 μg of VSV‐G, 5 μg of Δ8.9 and 8 μg of pLKO plasmids. After 36 h at 37 °C, virions were collected and opportunely diluted in fresh medium. Retroviral infections were performed as previously described (Di Giorgio *et al*., 2017).

### 2.4 RNA extraction and quantitative qRT‐PCR

Cells were lysed using Tri‐Reagent (Molecular Research Center, Cincinnati, OH USA). A total of 1 μg of total RNA was retrotranscribed using 100 U of Moloney murine leukemia virus reverse transcriptase (Invitrogen, Carlsbad, CA, USA). qRT‐PCR analyses were performed using Bio‐Rad CFX96 and SYBR green technology (Resnova, Roma, Italy). The data were analyzed by a comparative threshold cycle using the β2 microglobulin gene and hypoxanthine phosphoribosyltransferase 1 (HPRT) as normalizer genes. All reactions were done in triplicate.

### 2.5 Antibodies

Antibodies used were against MEF2D (BD Bioscience), H3K27ac (ab4729; Abcam, Cambridge, MA, USA). H3K27me3 (ab195477, Abcam) HDAC4 (Paroni *et al*., 2004), γH2AX (9718, Cell Signalling, Leiden, The Netherlands), TP53 (DO‐7; Dako, Santa Clara, CA, USA), LT SV40 (sc‐147, Santa Cruz), Lamin B1 (ab16048, Abcam), p21 (CP74, Sigma), RACK1 (sc‐17754, Santa Cruz, Dallas, TX, USA), GFP (Paroni *et al*., 2004).

### 2.6 Immunofluorescence and immunoblotting

Immunofluorescence and immunoblotting were performed as previously described (Di Giorgio *et al*., 2017). Briefly, cells were fixed with 3% paraformaldehyde and permeabilized with 0.1% Triton X‐100. Secondary antibodies were conjugated to Alexa Fluor 488, 546 and 633 (Molecular Probes, Eugene, OR, USA). Cells were imaged with a Leica confocal microscopy (LSM) SP2 and SP8. After SDS/PAGE and immunoblotting, cell lysates were incubated with primary antibodies. HPR‐conjugated secondary antibodies were from Sigma‐Aldrich and blots were developed with Super Signal West Dura (ThermoFisher Scientific, Waltham, MA, USA).

### 2.7 SA‐β‐gal assay

For SA‐β‐gal assay, cells were fixed in a solution of 2% formaldehyde/0.2% glutaraldehyde and stained with staining solution: 40 mm citric acid/Na phosphate buffer, 5 mm K_4_[Fe(CN)_6_]3H_2_O, 5 mm K_3_[Fe(CN)_6_], 150 mm sodium chloride, 2 mm magnesium chloride and 1 mg·mL^−1^ X‐gal (Debacq‐Chainiaux *et al*., 2009). Staining was performed for 16 h at 37 °C and cells were imaged under bright‐field microscope (Leica). To quantify SA‐β‐gal activity, the percentage of positively stained blue cells versus total cells were counted (300–400 cells in total were counted per each round).

### 2.8 Matrigel plug assay

A total of 1600 cells were suspended in a Matrigel solution (20 μL 0.1% FBS‐EMEM, 60 μL Matrigel) and plated on coverslips in 35‐mm tissue culture plates. After 30 min of incubation at 37 °C, cells were fed with EMEM–20% FBS. Following a 4‐day incubation, coverslips were fixed and processed for fluorescence analysis.

### 2.9 Transcriptome profiling and data analysis

Total RNA was isolated using Direct‐zol RNA mini prep (Zymo Research). Preparation and hybridization of cRNA samples were performed at Cogentech (Milan, Italy, https://www.cogentech.it/). Labeled cRNAs were hybridized on Affymetrix GeneChip Human Clariom S arrays. Differentially expressed genes (DEGs) were selected based on 1.5 cut‐off in the fold changes. Analysis was performed as previously described (Di Giorgio *et al*., 2013; Picco *et al*., 2014). Gene set enrichment analysis (GSEA) (Subramanian *et al*., 2005) and the MSigDB database (http://software.broadinstitute.org/gsea/index.jsp) (Liberzon *et al*., 2015) were used to investigate statistical association between genes modulated by HDAC4‐TM or RAS and genes perturbed by other conditions.

### 2.10 Statistics

For experimental data, a Student *t*‐test was used. A *P*‐value of 0.05 was chosen as the statistical limit of significance. Unless otherwise indicated, data in the figures are arithmetic means and standard deviations from at least three independent experiments: *P < 0.05, **P < 0.01, ***P < 0.005.

## 3 Results

### 3.1 Nuclear resident HDAC4 induces senescence in human fibroblasts (BJ‐TERT)

**Figure 1:**
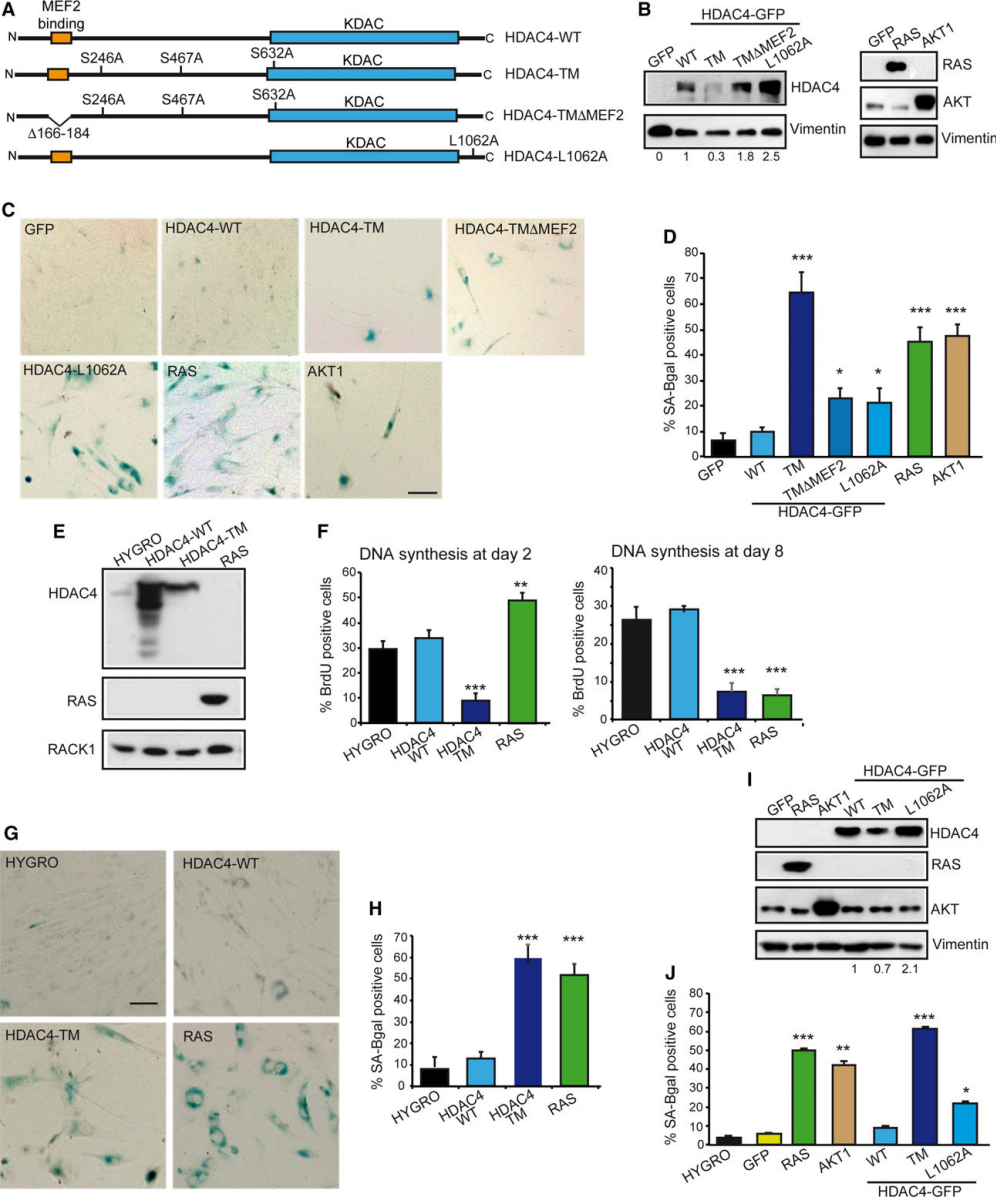
The HDAC4 expression in human fibroblasts. (A) Schematic representation of the different HDAC4 versions used in this study. Phosphorylated serine residues, binding sites for 14‐3‐3 proteins are indicated. (B) Immunoblot analysis using the indicated antibodies in BJ/TERT expressing the indicated transgenes. Vimentin was used as loading control. (C) Normal human diploid fibroblasts (BJ) expressing the telomerase catalytic subunit (TERT) infected with retrovirus expressing the indicated genes and stained for senescence‐associated β‐galactosidase (SA‐β‐gal) marker. Scale bar: 50 μm. (D) Quantitative analysis of SA‐β‐gal positivity from experiments described in (C). Data are expressed as means ± SD,* n* = 3. Student t‐test: *P < 0.05, ***P < 0.005. (E) Immunoblot analysis using the indicated antibodies in BJ/TERT expressing the indicated transgenes following treatment with 4‐OHT for 48 h. RACK1 was used as loading control. (F) Analysis of cells synthesizing DNA, as scored after BrdU staining at the indicated times. Data are expressed as means ± SD,* n* = 4. Student *t*‐test: **P < 0.01, ***P < 0.005. (G) BJ‐TERT expressing the indicated transgenes after 8 days of induction were stained for SA‐β‐gal activity. Scale bar: 50 μm. (H) Quantitative analysis of SA‐β‐gal positivity from experiments described in (G). Data are expressed as means ± SD,* n* = 4. ***P < 0.005. (I) Immunoblot analysis using the indicated antibodies in BJ/TERT expressing the indicated transgenes following treatment with doxycycline for 48 h. Vimentin was used as loading control. (J) Quantitative analysis of SA‐β‐gal positivity in BJ‐TERT cells expressing doxycycline‐inducible versions of the indicated transgenes after 8 days of induction. Data are expressed as means ± SD,* n* = 3. Student *t*‐test: *P < 0.05, **P < 0.01, ***P < 0.005.

To explore the contribution of HDAC4 to the transformation process we used skin‐derived BJ fibroblasts, immortalized with the telomerase catalytic subunit (TERT). Since HDAC4 is subjected to intense nuclear export following phosphorylation‐mediated 14‐3‐3 binding, we took advantage from a Ser/Ala mutated version in the three 14‐3‐3 binding sites (HDAC4‐TM). This mutant exhibits stronger repressive activity and is sufficient to transform NIH‐3T3 murine fibroblasts (Di Giorgio *et al*., 2013). Additional HDAC4 mutants tested were a nuclear resident form lacking the MEF2‐binding site (HDAC4‐TMΔMEF2) and a mutant in the nuclear export sequence (HDAC4‐L1062A), which similarly to the TM, accumulate in the nucleus (Fig. 1A) (Di Giorgio *et al*., 2013).

All HDAC4 versions were expressed in BJ‐TERT fibroblasts after retroviral infections. We also used well‐known oncogenes such as RAS (H‐RAS‐G12V) and AKT1 (myr‐AKT1), for comparison. Expression of the different transgenes was verified by immunoblot (Fig. 1B).

Non‐transformed human fibroblasts respond to the introduction of oncogenes by activating OIS (Astle *et al*., 2012; Kennedy *et al*., 2011; Serrano *et al*., 1997). Hence, we evaluated the presence of cells positive for SA‐β‐galactosidase activity (Fig. 1C). After selection, few cells were recovered when nuclear localized forms of HDAC4 were expressed. The few cells expressing HDAC4‐TM were positive on SA‐β‐gal staining, with a score comparable to RAS and AKT1 (Fig. 1D). Cells expressing two nuclear mutants of HDAC4 such as L1062A (Paroni *et al*., 2004) and TMΔMEF2 (Di Giorgio *et al*., 2013) were only moderately positive on SA‐β‐gal staining (Fig. 1D). Both mutants accumulate into the nucleus with a rate comparable to HDAC4‐TM but are less strongly bound to chromatin (Paroni *et al*., 2007). SA‐β‐gal positivity was not relevant in cells expressing HDAC4‐WT (which is largely cytoplasmic in BJ/TERT cells) or the negative control GFP (Fig. 1D).

To characterize the massive induction of senescence, we generated an estrogen receptor alpha (ER)‐inducible version of HDAC4‐TM, HDAC4‐WT and H‐RAS/G12V. HDAC4‐TM was expressed less compared with the wild type (Fig. 1E), possibly because of the proteasome‐dependent nuclear degradation of HDAC4 (Cernotta *et al*., 2011). Next, we analyzed cell proliferation 2 or 8 days after transgene induction (Fig. 1F). HDAC4‐TM triggered a rapid block in DNA synthesis. By contrast, RAS initially enhanced cell proliferation; only after 8 days of induction DNA synthesis was blocked. When the presence of SA‐β‐gal‐positive cells was investigated, only the induction of HDAC4‐TM and RAS stimulated SA‐β‐gal activity (illustrated in Fig. 1G and quantified in Fig. 1H). Finally, to corroborate these data and to exclude a contribution of tamoxifen to the process of senescence, we used a second inducible system. The different transgenes were efficiently induced by the doxycycline‐inducible system (Fig. 1I). The SA‐β‐gal staining confirmed that the induction of HDAC4‐TM triggers senescence with rates comparable to RAS and AKT1 (Fig. 1J). In summary, nuclear resident HDAC4 can trigger senescence in immortalized human fibroblasts. In contrast to RAS, an early proliferative block characterizes HDAC4‐TM‐induced senescence.

### 3.2 DNA damage in HDAC4‐TM‐induced senescence

**Figure 2:**
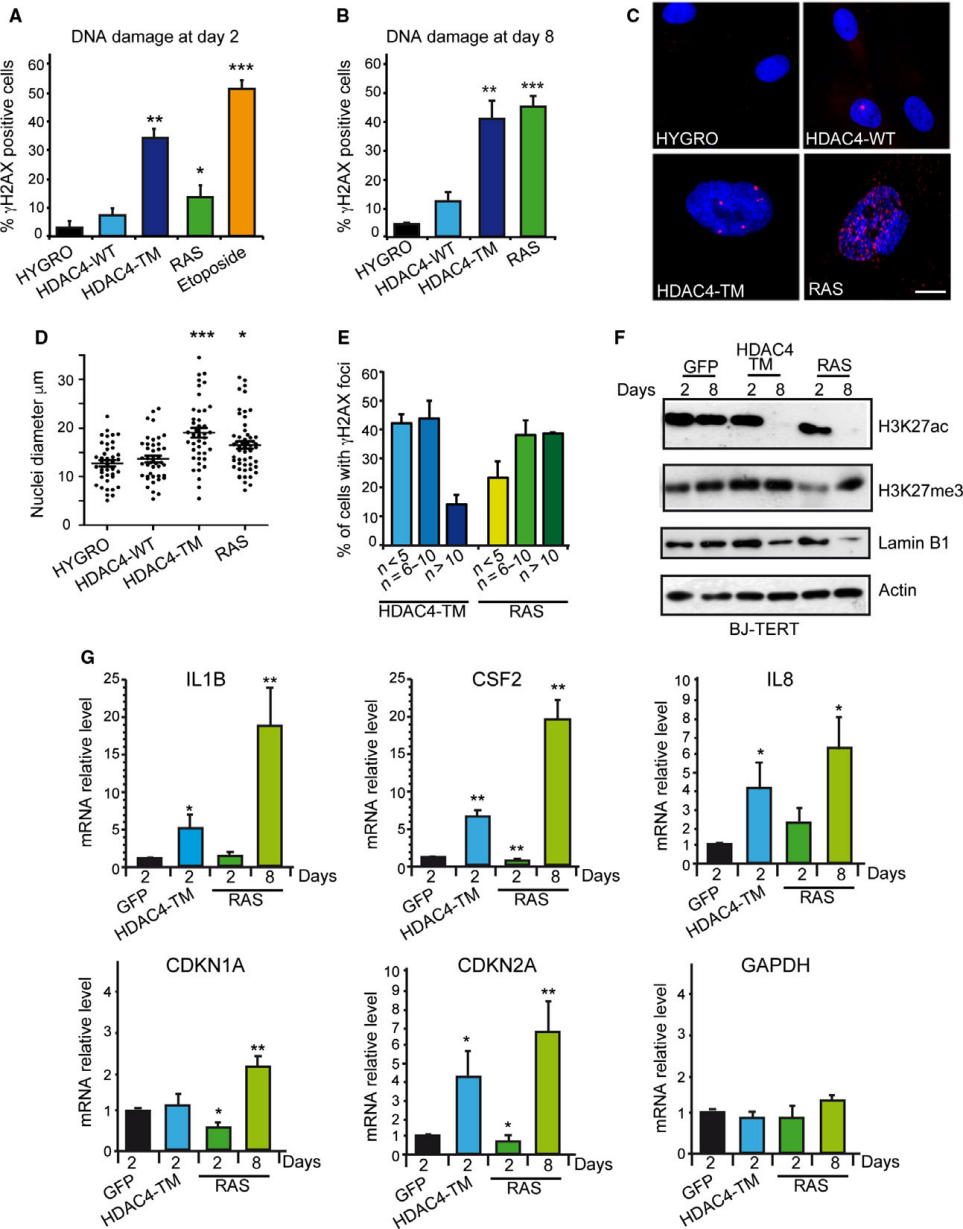
HDAC4‐induced senescence is characterized by DNA damage. (A) Quantitative analysis of the immunofluorescence staining for γH2AX after 2 days of induction of the indicated transgenes in BJ‐TERT cells. Data are expressed as means ± SD,* n* = 4. Student *t*‐test: *P < 0.05, **P < 0.01, ***P < 0.005. (B) Quantitative analysis of the immunofluorescence staining for γH2AX after 8 days of induction of the indicated transgenes in BJ‐TERT cells. Data are expressed as means ± SD,* n* = 4. Student *t*‐test: **P < 0.01, ***P < 0.005. (C) Immunofluorescence picture of γH2AX positivity after 8 days of induction of the indicated transgenes. Nuclei were stained with Hoechst 33342. Scale bar: 5 μm. (D) Quantitative analysis of nuclear dimension in BJ‐TERT cells following 8 days of induction of the different transgenes. Measures were obtained with imagej. At least 50 nuclei were scored for each condition. The means and the 1^st^ and 3^rd^ quartiles are indicated. *P < 0.05, ***P < 0.005. (E) Percentage of γH2AX‐positive cells with the indicated numbers of γH2AX foci in the nuclei of BJ‐TERT cells, after 8 days of expression of the indicated transgenes. (F) Immunoblot analysis using the indicated antibodies in BJ‐TERT cells expressing the different transgenes for 2 or 8 days. Actin was used as loading control. (G) mRNA expression levels of the indicated genes, as measured by qRT‐PCR in BJ/TERT cells expressing the different transgenes following treatment for the indicated days with 4‐OHT. Data are expressed as means ± SD,* n* = 3. Student *t*‐test: *P < 0.05, **P < 0.01.

OIS in RAS‐overexpressing cells is triggered by the accumulation of unrepaired damaged DNA caused by unscheduled DNA synthesis (Di Micco *et al*., 2006). Hence, we compared by time course the kinetics of accumulation of γH2AX‐positive cells in cells expressing HDAC4‐TM/ER, HDAC4‐WT/ER and H‐RAS‐G12V/ER. As early as 2 days after transgene induction, HDAC4‐TM‐expressing cells showed an accumulation of DNA damage (Fig. 2A). In contrast, in RAS‐expressing cells, DNA damage became consistent only after 8 days of induction with tamoxifen (Fig. 2B). A few γH2AX‐positive cells were also observed in the case of HDAC4‐WT (Fig. 2B). When immunofluorescence was analyzed in more detail, two phenotypes appeared evident: (a) nuclei in TM‐ and RAS‐expressing cells were larger than in control and HDAC4‐WT cells; (b) nuclei of TM cells showed fewer but bigger γH2AX‐positive foci than did RAS cells (Fig. 2C). Quantitative analysis confirmed this observation (Fig. 2D,E).

Next, we evaluated the epigenetic modifications induced by the different transgenes. The global levels of histone H3 lysine 27 acetylation (H3K27ac), a marker of open and transcriptional active chromatin, and of H3K27me3, a marker of facultative heterochromatin, were evaluated after 2 and 8 days of transgene induction. We also evaluated the levels of Lamin B1, which is down‐regulated during senescence (Freund *et al*., 2012). Whereas H3K27ac and Lamin B1 showed a similar behavior in TM and RAS cells, with a dramatic decrease at day 8 (Fig. 2F), the pattern of H3K27me3 was different between the two senescence responses. Only in RAS cells did a transient reduction occur at day 2, as recently observed (Ito *et al*., 2018).

Senescent cells produce and release a range of cytokines, chemokines and proteases in the extracellular environment: the senescence‐associated secretory phenotype (SASP) (Coppé *et al*., 2008). Hence, we compared the kinetics of appearance of senescence and SASP after HDAC4‐TM or RAS induction by analyzing the expression levels of SASP components (*IL1B, IL8* and *CSF2*) and those of the CDK inhibitors *CDKN1A* and *CDKN2A*, key regulators of the senescent response. QRT‐PCR analysis proved that HDAC4‐TM anticipated the appearance of senescence with respect to RAS (Fig. 2G) in terms of SASP and *CDKN2A* induction. In RAS‐expressing cells this response was only evident after 8 days of induction.

### 3.3 HDAC4‐induced senescence depends on TP53 activation

**Figure 3:**
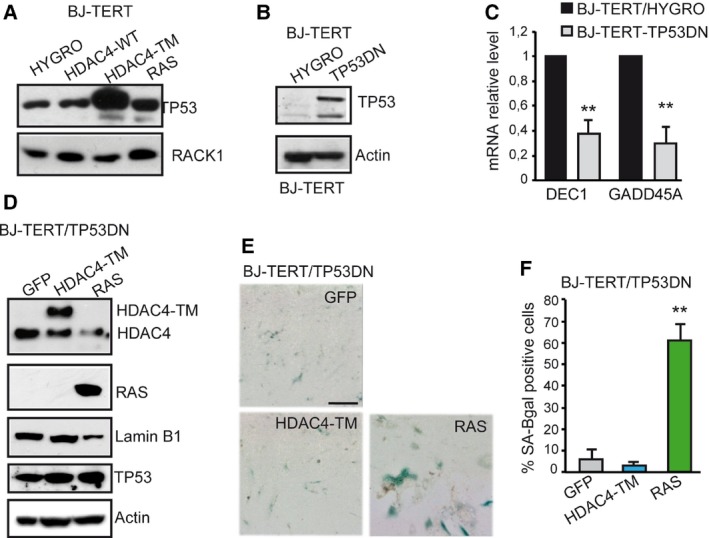
The HDAC4‐induced senescence is characterized by TP53 activation. (A) TP53 levels in BJ‐TERT cells expressing the indicated transgenes for 8 days. RACK1 was used as loading control. (B) Immunoblot analysis proving the generation of BJ‐TERT cells expressing the DN TP53. Actin was used as loading control. (C) qRT‐PCR analysis evaluating the expression of two TP53 target genes in the different BJ‐TERT cell lines as indicated. mRNA levels are relative to BJ‐TERT/HYGRO cells. Data are expressed as means ± SD,* n* = 3. **P < 0.01. (D) Immunoblot analysis evaluating the levels of indicated proteins in BJ‐TERT cells after 8 days of 4‐OHT treatment. The HDAC4‐TM is expressed as fusion with GFP. Actin was used as loading control. (E) BJ‐TERT/TP53DN expressing the indicated transgenes and stained for SA‐β‐gal activity. Scale bar: 50 μm. (F) Quantitative analysis of SA‐β‐gal positivity from experiments described in (F). Data are expressed as means ± SD,* n* = 4. **P < 0.01.

The induction of DNA damage in TM‐expressing cells prompted us to investigate the contribution of TP53. Immunoblot analysis performed after 8 days of transgene induction demonstrated a strong up‐regulation of TP53 levels in TM cells (Fig. 3A). To investigate the contribution of TP53 in TM‐induced senescence, we generated BJ‐TERT cells expressing TP53 mutant R175H (Fig. 3B). This mutant is frequently found in human cancers and acts as a dominant negative (TP53DN) (Gualberto *et al*., 1998). The DN effect was shown on the TP53‐target genes *DEC1* and *GADD45A* (Fig. 3C). Subsequently, we generated BJ‐TERT/TP53 cells expressing HDAC4‐TM, RAS or GFP as control. Immunoblot analysis confirmed the expression of the different transgenes and showed that Lamin B1 was not down‐regulated in TM cells, thus suggesting the escape from senescence (Fig. 3D). SA‐β‐gal activity (Fig. 3E) and the relative quantitative analysis (Fig. 3F) confirmed the failure of TM in triggering senescence, once the TP53 response was blunted. In contrast, in RAS‐expressing cells, suppression of TP53 activities was not sufficient to block the occurrence of senescence (Fig. 3E,F), as previously observed (Serrano *et al*., 1997).

These experiments demonstrate that HDAC4‐TM triggers senescence, which is markedly different from RAS, since it is characterized by: (a) an early proliferative block, (b) an early and peculiar induction of DNA damage and (c) a strong TP53‐dependency.

### 3.4 Down‐regulation of MEF2 transcription is sufficient to promote senescence

**Figure 4:**
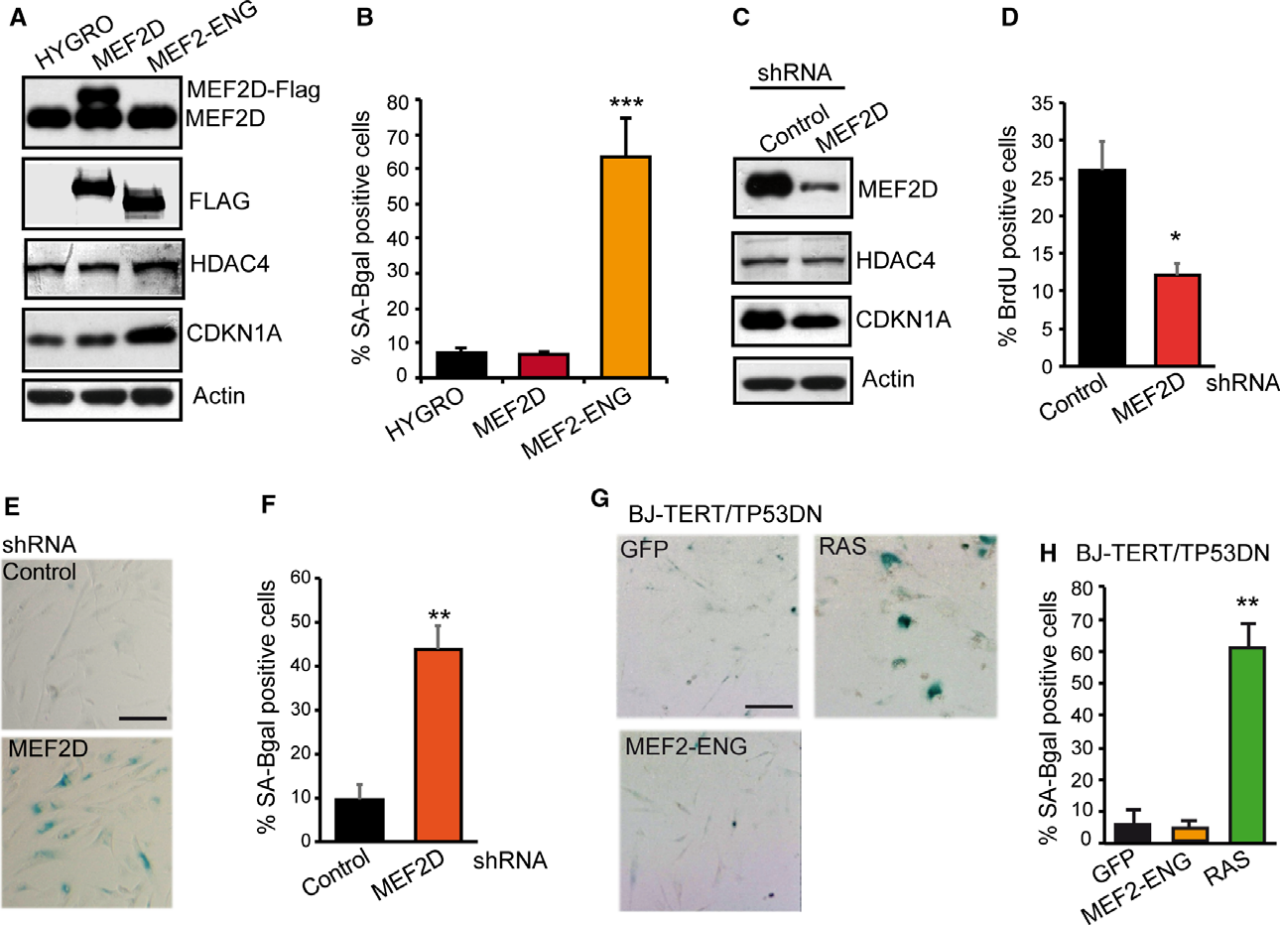
Myocyte enhancer factor transcriptional activity counteracts senescence. (A) Immunoblot analysis evaluating the levels of the indicated proteins in BJ‐TERT cells. MEF2D and MEF2‐ENG contain a Flag epitope. Actin was used as loading control. (B) Quantitative analysis of SA‐β‐gal positivity in BJ‐TERT cells expressing the indicated transgenes. (C) Immunoblot analysis of MEF2D, HDAC4 and CDKN1A levels in BJ‐TERT cells expressing the control shRNA or a shRNA against MEF2D. shRNA was delivered by lentiviral infection. Actin was used as loading control. (D) Analysis of DNA synthesis, as scored after BrdU staining in BJ‐TERT cells expressing the two shRNA. Data are expressed as means ± SD,* n* = 3. *P < 0.05. (E) BJ‐TERT expressing the indicated shRNA were stained for SA‐β‐gal activity. Scale bar: 50 μm. (F) Quantitative analysis of SA‐β‐gal positivity from experiments described in (E). Data are expressed as means ± SD,* n* = 4. **P < 0.01. (G) BJ‐TERT/TP53 expressing the indicated transgene were stained for SA‐β‐gal activity. Scale bar: 50 μm. (H) Quantitative analysis of SA‐β‐gal positivity from experiments described in (G). Data are expressed as means ± SD,* n* = 4. ***P < 0.005.

HDAC4 can interact with several proteins, among which MEF2 TFs represent important partners (Clocchiatti *et al*., 2013a). Since HDAC4‐TM deleted in the MEF2‐binding region triggers senescence less potently compared with HDAC4‐TM (Fig. 1C), we hypothesized that the induction of senescence could be partially due to the repression of these TFs. To clarify this point, we generated BJ‐TERT cells expressing MEF2‐ENG, a repressive version of these TFs in which the C‐terminal MEF2 activation domain is substituted by the Engrailed repressor domain (Arnold *et al*., 2007). Cells overexpressing MEF2D or HYGRO were used as controls (Fig. 4A). Appearance of senescence was clearly observed in BJ‐TERT‐MEF2/ENG cells but not in the controls (Fig. 4B). To alternatively down‐regulate the expression of MEF2‐target genes, we silenced the expression of MEF2D using a shRNA (Fig. 4C). MEF2D downregulation triggered a senescence response, characterized by a strong impairment in S‐phase entry (Fig. 4D) and the accumulation of SA‐β‐gal‐positive cells (Fig. 4E and quantitative analysis in Fig. 4F). Finally, similarly to HDAC4‐TM, senescence in response to MEF2 transcriptional repression is TP53‐dependent. BJ‐TERT‐TP53DN cells expressing RAS entered into senescence, whereas SA‐β‐gal‐positive cells were not detected when MEF2‐ENG was introduced (Fig. 4G and quantitative analysis in Fig. 4H).

### 3.5 HDAC4‐mediated repression cooperates with SV40‐LT in transforming human fibroblasts

To prove that BJ/TERT cells expressing the nuclear version of HDAC4 enter senescence as part of a protective response against an oncogenic dysfunction, it is imperative to prove that HDAC4 provides some transforming properties. Since anchorage‐independent growth is a clear marker of cellular transformation, we initially decided to monitor this feature in BJ/TERT/TP53DN/HDAC4‐TM cells that escape senescence. We also analyzed the effect of the overexpression of HDAC4‐WT, MEF2‐ENG and RAS.

**Figure 5:**
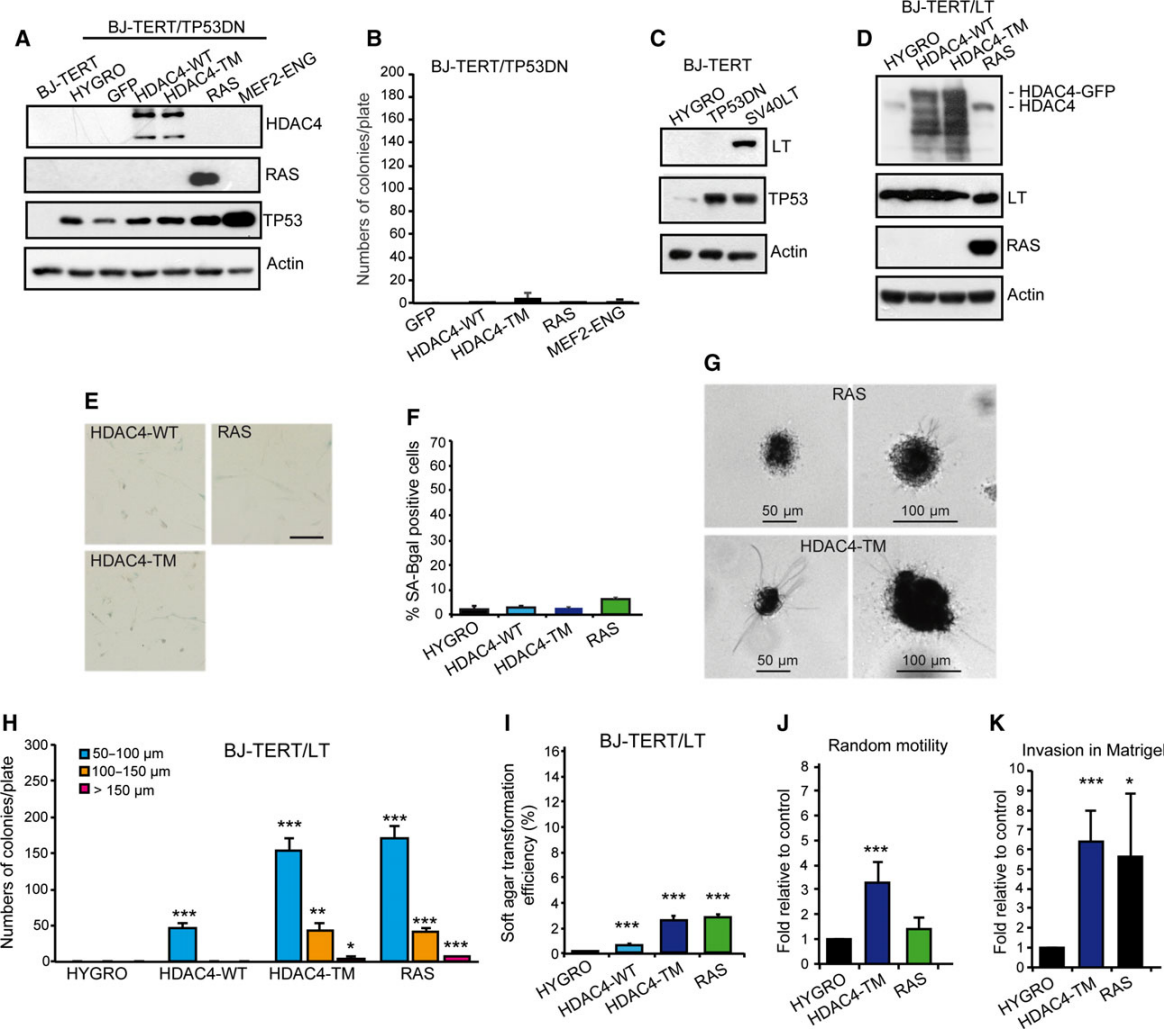
The HDAC4‐TM cooperates with SV40 LT in sustaining a transformed phenotype. (A) Immunoblot analysis of indicated proteins as expressed in BJ‐TERT/TP53DN cells infected with retrovirus encoding the indicated genes. HDAC4 was expressed as fusion with GFP and the recombinant proteins detected with an anti‐GFP antibody. Actin was used as loading control. (B) Growth in soft agar of the BJ‐TERT/TP53 cells expressing the indicated transgene. After staining with MTT, foci with diameter > 50 μm were counted. Data are expressed as means ± SD,* n* = 3. Student *t*‐test: *P < 0.05, **P < 0.01, ***P < 0.005. (C) Immunoblot analysis of indicated proteins as expressed in BJ‐TERT cells infected with retrovirus encoding the indicated genes. Actin was used as loading control. (D) Immunoblot analysis of indicated proteins as expressed in BJ‐TERT/LT cells infected with retrovirus encoding the indicated genes. HDAC4 was expressed as fusion with GFP and the recombinant proteins detected with an anti‐GFP antibody Actin was used as loading control. (E) BJ‐TERT/LT cells expressing the indicated transgenes were stained for SA‐β‐gal activity. Scale bar: 50 μm. (F) Quantitative analysis of SA‐β‐gal positivity from experiments described in (E). Data are expressed as means ± SD,* n* = 3. Student *t*‐test: *P < 0.05, **P < 0.01, ***P < 0.005. (G) Representative images of MMT positive soft agar foci of BJ‐TERT/LT cells expressing HDAC4‐TM or RAS. Scale bars are indicated. (H) Quantitative analysis of soft agar foci of BJ‐TERT/LT cells expressing the indicated transgenes grouped for foci dimensions related to (G). Data are expressed as means ± SD,* n* = 4. Student *t*‐test: *P < 0.05, **P < 0.01, ***P < 0.005. (I) Soft agar transformation efficiency represents the total number of foci generated by BJ‐TERT/LT cells expressing the indicated transgenes, divided by the total number of seeded cells. Data are expressed as means ± SD,* n* = 4. ***P < 0.005. (J) Motility properties of BJ‐TERT/LT cells expressing RAS, HDAC4‐TM or HYGRO as control. Data are expressed as means ± SD,* n* = 3. ***P < 0.005. (K) Invasive properties, as measured by Matrigel invasion assay of BJ‐TERT/LT cells expressing RAS, HDAC4‐TM or Hygro as control. Data are expressed as means ± SD,* n* = 3. *P < 0.05, ***P < 0.005.

Expression of the different transgenes was verified by immunoblot (Fig. 5A) and soft agar assays were performed to evaluate the anchorage‐independent growth. In none of the engineered cell lines was growth in soft agar detectable (Fig. 5B). This result indicates that HDAC4‐TM‐ or MEF2‐mediated gene repression cannot sustain anchorage‐independent growth once senescence is suppressed by TP53 mutations.

The SV40 large T antigen (LT) can inactivate two major tumor suppressor genes, p53 and retinoblastoma protein (pRb) (Hahn *et al*., 2002). In several experiments LT has proved to cooperate in the transformation of human cells (Hahn *et al*., 1999). Hence, we generated BJ‐TERT/LT cells (Fig. 5C) to evaluate oncogenic cooperation with HDAC4‐TM, HDAC4‐WT and RAS as a control (Fig. 5D). In contrast to TP53DN, LT expression suppressed OIS by RAS (Fig. 5E and quantitative analysis in Fig. 5F). Growth in soft agar was performed and the appearance of foci was scored 21 days later. We also evaluated the size distribution of the colonies (Fig. 5G) using already published criteria (Cifone and Fidler, 1980). Small colonies with a 50‐μm diameter or less were frequently observed in RAS‐ and HDAC4‐TM‐expressing cells (Fig. 5H). Rarely, larger colonies with a diameter of 100–150 μm were present. Interestingly, foci of small dimensions were also observed when TERT/LT/HDAC4‐WT were co‐expressed. Analysis of the transformation efficiency confirmed that HDAC4‐TM and RAS showed comparable activities. A much weaker transforming effect was observed for HDAC4‐WT (Fig. 5I).

To confirm the induction of a transformed phenotype we evaluated random motility and invasiveness using the Matrigel invasion assay. Figure 5J shows that only HDAC4‐TM enhanced random cell motility but both RAS and HDAC4‐TM strongly promoted invasiveness in TERT/LT human cells (Fig. 5K).

**Figure 6:**
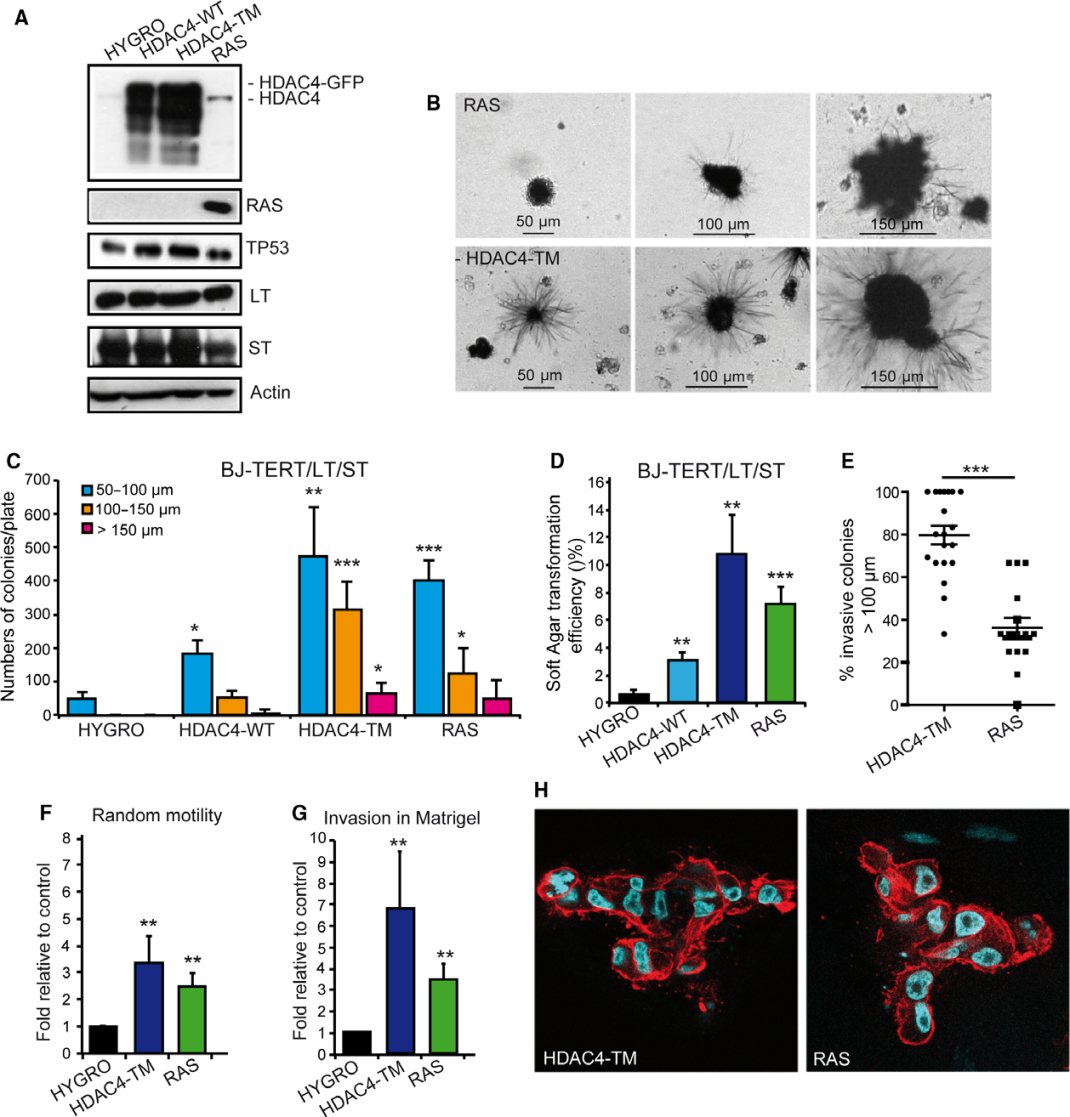
The HDAC4‐TM cooperates with SV40 LT in sustaining a transformed phenotype. (A) Immunoblot analysis of indicated proteins as expressed in BJ‐TERT/LT/ST cells infected with retrovirus encoding the indicated genes. HDAC4 was expressed as fusion with GFP and the recombinant proteins detected with an anti‐GFP antibody. Actin was used as loading control. (B) Representative images of MMT‐positive soft agar foci of BJ‐TERT/LT/ST cells expressing HDAC4‐TM or RAS. Scale bars are indicated. (C) Quantitative analysis of soft agar foci of BJ‐TERT/LT/ST cells expressing the indicated transgenes grouped for foci dimensions related to Fig. 6B. Data are expressed as means ± SD,* n* = 4. Student *t*‐test: *P < 0.05, **P < 0.01, ***P < 0.005. (D) Soft agar transformation efficiency represents the total number of foci generated by BJ‐TERT/LT/ST cells expressing the indicated transgenes divided by the total number of seeded cells. Data are expressed as means ± SD,* n* = 4. **P < 0.01, ***P < 0.005. (E) Percentage of invasive soft agar foci. Foci were scored for the presence of cells showing extensive growth in the surrounding agar. The means and the 1st and 3rd quartiles are indicated. *n* = 19 (HDAC4‐TM) and *n* = 15 (RAS). ***P < 0.005. (F) Motility properties of BJ‐TERT/LT/ST cells expressing RAS, HDAC4‐TM or Hygro as control. Data are expressed as means ± SD,* n* = 3. Student *t*‐test: **P < 0.01. (G) Invasive properties, as measured by Matrigel invasion assay of BJ‐TERT/LT/ST cells expressing RAS, HDAC4‐TM or Hygro as control. Data are expressed as means ± SD,* n* = 3. **P < 0.01. (H) Confocal images representing the equatorial section of BJ‐TERT/LT/ST cells expressing RAS or HDAC4‐TM grown in Matrigel plugs. Nuclei were visualized using Hoechst 33342 and actin filaments with phalloidin‐Alexa 546.

To have a full transformation of human fibroblasts, both the LT and the SV40 small T antigen (ST) must be co‐expressed with TERT and RAS (Hahn *et al*., 2002). Hence, we generated human fibroblasts expressing the combination of TERT/LT/ST together with HDAC4‐WT, HDAC4‐TM or RAS as a positive control of transformation. Immunoblot analysis verified the expression of the different transgenes (Fig. 6A). Next, soft agar assay was performed and after 21 days, colonies were stained with MTT and foci were counted and grouped for dimensions as exemplified (Fig. 6B). Quantitative analysis proved that the expression of the ST increased the number of foci and particularly their dimensions (Fig. 6C) as well as the transformation efficiency (compare Fig. 6D with Fig. 5I). Interestingly, cells expressing HDAC4‐TM frequently show extensive invasion and some branching into the agar (Fig. 6B). Quantitative analysis confirmed the invasive properties of foci generated by HDAC4‐TM (Fig. 6E). Here, again, growth in soft agar was also observed when HDAC4‐WT was expressed, although with reduced efficiency compared with HDAC4‐TM (Fig. 6C,D).

To confirm the invasive properties of TERT/LT/ST/HDAC4‐TM cells we performed the Matrigel invasion assay. Random cell motility and invasion were measured (Fig. 6F,G). These experiments confirmed the strong invasive behavior of HDAC4‐TM‐expressing cells.

Finally, we analyzed TERT/LT/ST cells expressing HYGRO, RAS and HDAC4‐TM in three‐dimensional Matrigel plugs. Only cells expressing RAS or HDAC4‐TM exhibited robust growth and invasion into the matrix (Fig. 6H).

### 3.6 Common and specific transcriptomic adaptations characterize RAS and HDAC4‐TM transformed cells

**Figure 7:**
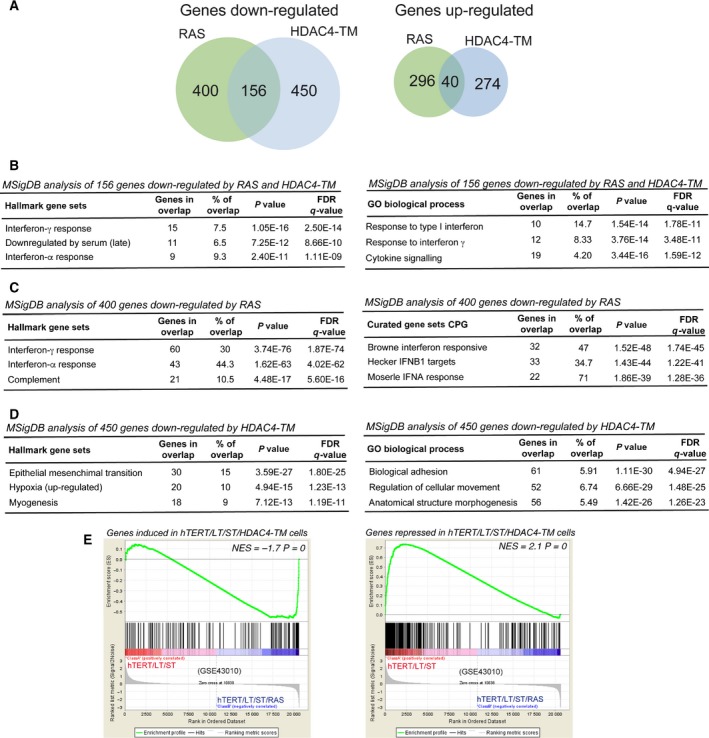
Gene expression profiles of RAS and HDAC4‐TM transformed human fibroblasts. (A) Pie‐chart indicating the number of genes significantly up‐ and down‐regulated in TERT/LT/ST/RAS and TERT/LT/ST/HDAC4‐TM cells compared with TERT/LT/ST/HYGRO cells. (B) GSEA for the 156 genes commonly repressed by RAS and HDAC4‐TM using the hallmark and the GO/biological process MSigDB gene sets. (C) GSEA for the 400 genes specifically repressed by RAS using the hallmark and the curated (CPG) MSigDB gene sets. (D) GSEA for the 450 genes specifically repressed by HDAC4‐TM using the hallmark and the GO/biological process MSigDB gene sets. (E) GSEA plots showing significant enrichment for HDAC4‐regulated genes in a BJ transformation model (GSE43010), considering both HDAC4‐positively regulated genes (left) and HDAC4‐repressed genes (right).

To gain insight into the molecular mechanisms responsible for the RAS‐ and HDAC4‐TM‐ mediated oncogenic transformation, we performed gene expression profile studies. The transcriptomes of TERT/LT/ST/HYGRO, TERT/LT/ST/RAS and TERT/LT/ST/HDAC4‐TM were compared. DEGs were analyzed relative to TERT/LT/ST/HYGRO cells. Figure 7A shows that RAS and HDAC4‐TM influence the expression of a comparable number of genes (respectively 892 and 920). In both cases, the number of repressed genes is more than the double that of the induced ones. Moreover, HDAC4‐TM and RAS have a larger pool (15.5%, *n* = 156 genes) of all the repressed genes in common, in contrast to 6.5% (*n* = 40) of up‐regulated genes. Since HDAC4 is a transcriptional repressor, we focused our analysis on the repressed genes. The 156 commonly repressed genes turned out to be highly enriched for elements of the interferon (IFN) pathways (Fig. 7B). In addition and in agreement with the transformed state of these cells, a subset of the commonly silenced genes belong to the category of genes repressed by serum/growth factors (Fig. 7B). Next, we evaluated RAS‐ and HDAC4‐TM‐specific repressive signatures. IFN and, in general, inflammatory signatures were prevalent among genes (*n* = 400) specifically repressed by RAS (Fig. 7C). In contrast, genes that are specifically repressed by HDAC4‐TM are involved in more heterogeneous regulative processes, including the epithelial‐mesenchymal transition, the hypoxia response and differentiation/morphogenesis (Fig. 7D). The high number of genes regulating cell adhesion and migration, as disclosed by the Gene Ontology analysis, could explain the peculiar phenotype of the soft agar colonies generated by TERT/LT/ST/HDAC4‐TM cells (Fig. 6).

Finally, the comparison of the transcriptomic profiles indicated that the transformation phenotype elicited by HDAC4‐TM in TERT/LT/ST cells resembles adaptations in gene expression previously described in other models of human fibroblast transformation (Fig. 7E).

## 4 Discussion

In this manuscript we used human fibroblasts to evaluate the oncogenic cooperative properties of HDAC4. The transforming potential of class IIa HDACs has been shown in different murine models (Di Giorgio *et al*., 2013; Gil *et al*., 2016; Lei *et al*., 2017; Peruzzo *et al*., 2016; Rad *et al*., 2010). However, since there are substantial interspecies differences in the biology of cell transformation (Boehm *et al*., 2005), we decided to verify the oncogenic properties of HDAC4 in human cells.

The combined expression of HDAC4‐TM with the viral oncogene LT and much more strongly with LT and ST viral oncogenes can sustain growth in soft agar, similarly to RAS oncogene. Although at reduced levels, HDAC4‐WT can also promote anchorage‐independent growth when co‐expressed with LT and ST.

The ability of LT to cooperate with HDAC4 strongly indicates that inactivation of the pRb and TP53 tumor suppressor pathways is required to unleash the HDAC4‐transforming potential. However, in the absence of ST, the growth is not robust and foci are less frequently observed and of small size. This behavior was previously reported in the case of RAS (Hahn *et al*., 2002).

ST is required to increase dramatically the anchorage‐independent growth of both HDAC4 and RAS. ST‐transforming activities rely on the binding and inhibition of some isoforms of the serine‐threonine phosphatase, PP2A (Hahn *et al*., 2002). PP2A can dephosphorylate and regulate several targets, thus making it difficult to hypothesize which pathway could synergize with HDAC4. Recently, it has been shown that the PAK1‐YAP axis can mediate ST‐transforming activity (Nguyen *et al*., 2014). Interestingly, a contribution of HDAC4 in mediating YAP‐repressive activity has been reported (Wang *et al*., 2014).

Transcriptomic analysis has revealed that HDAC4‐TM and RAS trigger the transformation processes via both common and peculiar adaptive responses. Repression of genes marking the IFN responses was commonly found in RAS‐ and HDAC4‐TM‐transformed cells. IFN and inflammatory signaling were highly enriched also among genes specifically repressed by RAS. Although it is well known that RAS and its downstream elements suppress the IFN‐induced antiviral responses and favor virus spreading (Battcock *et al*., 2006; Christian *et al*., 2009; Noser *et al*., 2007), how this repressive wave could impact on the *in vitro* transformation process is less clear (Christian *et al*., 2012). Gene signatures specifically influenced by HDAC4‐TM are more heterogeneous and involve adaptation to hypoxia, adhesion, motility and differentiation processes. It is possible that RAS more potently suppresses the IFN responses compared with HDAC4‐TM, which instead represses additional pathways.

The ability of HDAC4‐TM to regulate genes involved in adhesion and motility was confirmed in the morphological analysis of soft agar foci as well as in the results obtained with Matrigel invasion and evasion assays. These results indicate that HDAC4‐expressing cells exhibit a strong invasive phenotype, further supported by previous studies on the invasive, migrating and metastatic activities of class IIa HDACs (Cao *et al*., 2017; Cernotta *et al*., 2011; Di Giorgio *et al*., 2013; Fabian *et al*., 2016; Mottet *et al*., 2007).

Normal cells in response to oncogenic signals enter into senescence, a state of irreversible/permanent growth arrest that prevents cells from undergoing further cell divisions, defined as OIS (Serrano *et al*., 1997). Activation of OIS depends on the pRB and/or TP53 tumor suppressor pathways (Serrano *et al*., 1997). We have proved that HDAC4‐TM, in TERT‐immortalized human fibroblasts, can activate senescence. This senescent response can be also triggered by other class IIa HDACs such as HDAC7, when localized into the nucleus (Supporting Information Fig. S1). Since the expression of HDAC4‐TM in the opportune genetic environment (LT/ST co‐expression) can transform cells, and since the senescent response is p53‐dependent, we can define senescence triggered by HDAC4‐TM as OIS. However, OIS induced by RAS cannot be reversed by simply blocking TP53 activity, but requires the suppression of pRB, possibly through the CDK inhibitor p16 (Serrano *et al*., 1997). The difference between HDAC4‐TM and RAS can be appreciated also at the earliest stages of their induction. RAS triggers hyperproliferation and S‐phase‐associated DNA damage response (DDR). The oncogene‐dependent increase in proliferation leads to accumulation of incomplete replication intermediates, resulting in DNA damage and activation of the DDR (Di Micco *et al*., 2006).

In contrast, HDAC4‐TM triggers suddenly growth arrest, senescence and SASP, which could be caused by the rapid activation of TP53. The absence of the hyperproliferative response correlates with the failure to trigger H3K27 global demethylation, as observed in RAS‐expressing cells.

### 4.1 How can HDAC4‐TM trigger TP53 stabilization and senescence?

Induction of DNA damage, marked by γH2AX positivity, was observed. In contrast to RAS, the number of γH2AX spots per cell was reduced in HDAC4‐TM cells. Hence, the induction of DNA damage and TP53 activation seems to involve different pathways compared with the replication stress induced by RAS. Previous reports have described correlations between HDAC4 and the DNA damage response, and also with TP53 regulation (Cadot *et al*., 2009; Marampon *et al*., 2017). Unfortunately, these preliminary observations have not led to further studies and currently the correlations between class IIa HDACs and the DNA damage response remain unknown.

Although RAS‐induced OIS is among the foremost pathways, alternative OIS pathways exist. PI3K/AKT‐induced senescence is characterized by TP53 and CDKN1A induction in the absence of overt DNA damage. mTORC1‐dependent regulation of TP53 translation and stabilization of TP53 protein, as a result of MDM2 nucleolar localization and inactivation, is the mechanism activated in this senescent response (Astle *et al*., 2012; Kennedy *et al*., 2011). Importantly, similarly to HDAC4, AKT promotes a rapid proliferative arrest in the absence of hyperproliferation (Astle *et al*., 2012; Kennedy *et al*., 2011).

Interestingly, in certain cancer types, particularly in leiomyosarcomas, class IIa HDACs and PI3K/AKT can regulate common genetic programs that are under the influence of MEF2 TFs. Class IIa HDAC binds MEF2 on target promoters and favors the establishment of a closed chromatin configuration (Di Giorgio *et al*., 2017). In contrast, PI3K/AKT can trigger polyubiquitylation and degradation of certain MEF2 isoforms (Di Giorgio *et al*., 2015). As MEF2s are possible common targets of these pathways, it is important to underline that the repression of MEF2 TFs is required for the OIS response triggered by HDAC4 and that the knock‐down of MEF2D is sufficient to trigger senescence.

## 5 Conclusions

Our results provide further evidence concerning the contribution, as cooperating oncogenes, of class IIa HDACs in human cancer. This finding is encouraging for further investigations aimed at discovering and evaluating inhibitors of these epigenetic regulators as anticancer drugs.

## Authors’ contributions

CB and EDG designed the study. HP and EDG performed the experiments. CB wrote and revised the manuscript. All authors read and approved the final manuscript.

## Supporting information


**Fig. S1.** HDAC7‐S/A triggers senescence similarly to HDAC4‐TM.
