# Supplementary figures and images for: Unscheduled HDAC4 repressive activity in human fibroblasts triggers TP53‐dependent senescence and favors cell transformation
Source: Mol Oncol. 2018 Nov 14;12(12):2165–81. doi: 10.1002/1878-0261.12392 (PMC6275271; doi:10.1002/1878-0261.12392)

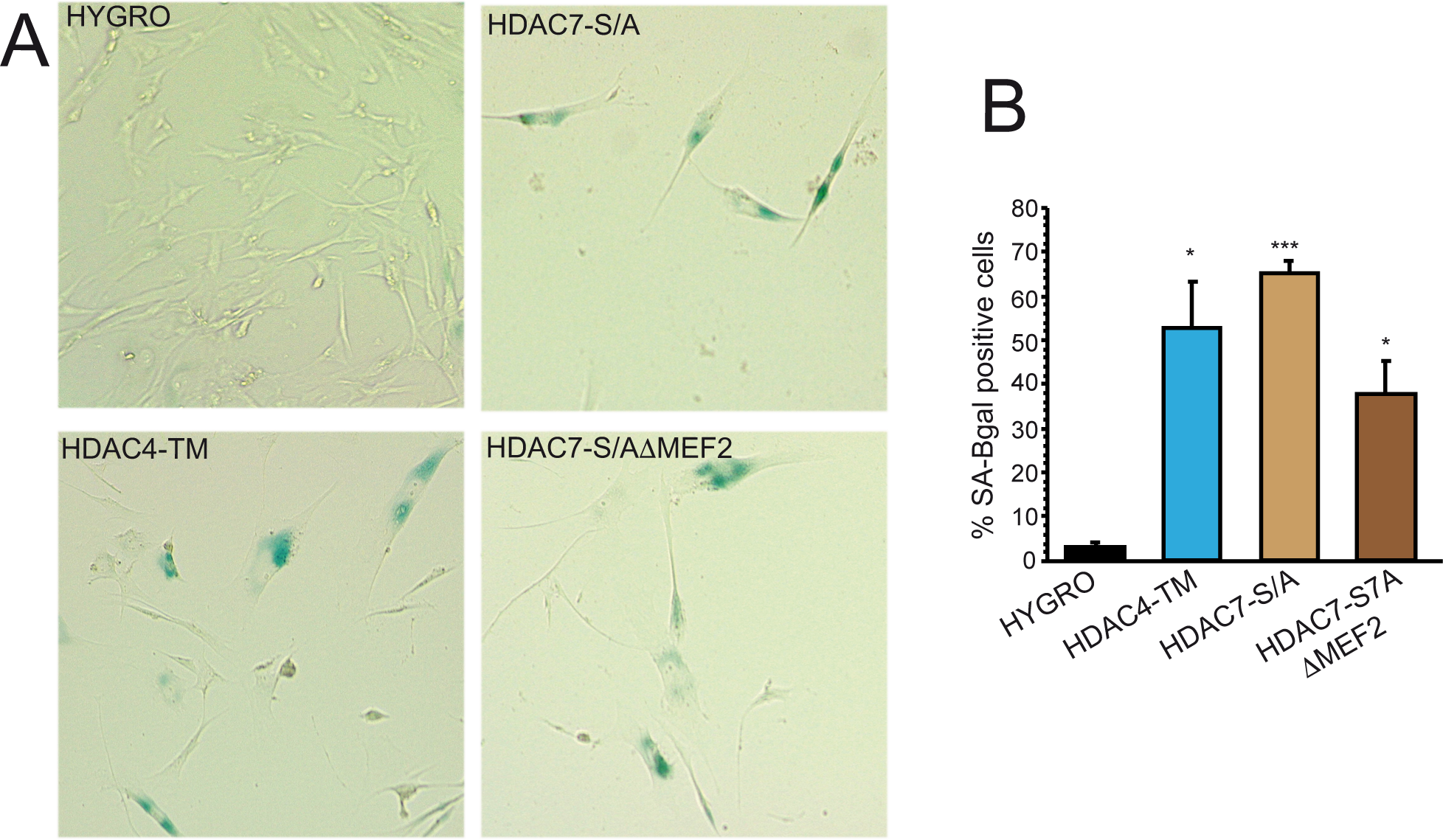

Supplement: Supplementary file 1 — Fig. S1. HDAC7‐S/A triggers senescence similarly to HDAC4‐TM. [file MOL2-12-2165-s001.tif]
